# Emerging Electrolyte-Gated Transistors: Materials, Configuration and External Field Regulation

**DOI:** 10.3390/ma18184320

**Published:** 2025-09-15

**Authors:** Dihua Tang, Wen Deng, Xin Yan, Jean-Jacques Gaumet, Wen Luo

**Affiliations:** 1 Department of Physics, School of Physics and Mechanics, Wuhan University of Technology, Wuhan 430070, China; 2 State Key Laboratory of Advanced Technology for Materials Synthesis and Processing, Wuhan University of Technology, Wuhan 430070, China; 3 Laboratoire de Chimie et Physique: Approche Multi-échelles, des Milieux Complexes (LCP-A2MC), Institut Jean Barriol, Université de Lorraine, 57070 Metz, France

**Keywords:** electrolyte-gated transistors, electrolyte materials, device architectures, multi-field regulation, synaptic devices

## Abstract

Electrolyte-gated transistors (EGTs) have emerged as a highly promising platform for neuromorphic computing and bioelectronics, offering potential solutions to overcome the limitations of the von Neumann architecture. This comprehensive review examines recent advancements in EGT technology, focusing on three critical dimensions: materials, device configurations, and external field regulation strategies. We systematically analyze the development and properties of diverse electrolyte materials, including liquid electrolyte, polymer-based electrolytes, and inorganic solid-state electrolytes, highlighting their influence on ionic conductivity, stability, specific capacitance, and operational characteristics. The fundamental operating mechanisms of EGTs and electric double layer transistors (EDLTs) based on electrostatic modulation and ECTs based on electrochemical doping are elucidated, along with prevalent device configurations. Furthermore, the review explores innovative strategies for regulating EGT performance through external stimuli, including electric fields, optical fields, and strain fields/piezopotentials. These multi-field regulation capabilities position EGTs as ideal candidates for building neuromorphic perception systems and energy-efficient intelligent hardware. Finally, we discuss the current challenges such as material stability, interfacial degradation, switching speed limitations, and integration density. Furthermore, we outline future research directions, emphasizing the need for novel hybrid electrolytes, advanced fabrication techniques, and holistic system-level integration to realize the full potential of EGTs in next-generation computing and bio-interfaced applications.

## 1. Introduction

The von Neumann architecture, proposed by mathematician John von Neumann in 1945, forms the foundation of modern computing [1]. While its adaptability and scalability allow computers to perform diverse tasks by altering stored programs, the explosive growth of Artificial Intelligence (AI) and the Internet of Things (IoT) has generated unprecedented volumes of data, presenting significant challenges for rapid information processing. A fundamental limitation of the traditional von Neumann architecture is the shared pathway for data storage and computation, hindering the efficient handling of massive datasets. Critical computational constraints including limited parallelization, excessive power demands, and elevated latency stem from the dual pressures of Moore’s Law culmination and persistent von Neumann bottlenecks [2,3,4,5,6]. Consequently, there exists an urgent need to develop novel devices and innovative computing paradigms to meet escalating demands for data storage and processing [7].

Memristors [5,8], representing a leading approach for memory–processor integration, have garnered significant attention. Their appeal stems from non-volatile resistive switching characteristics, low power consumption, rapid storage capability, in-memory computing potential, and the capacity to overcome limitations inherent in the von Neumann architecture [3,5,8]. Neuromorphic devices, capable of emulating neuronal and synaptic functions, hold immense promise for hardware-based neuromorphic platforms and constitute a transformative computing paradigm [9,10,11,12,13]. Among these, Electrolyte-Gated Transistors (EGTs) have emerged as one of the most promising candidates for realizing artificial neurons and synapses [14,15,16,17].

EGTs utilize ionic conductors as the gate dielectric [18,19,20]. Their unique capacitive coupling mechanism arises from ion/electron interfacial coupling, enabling rich physicochemical effects, which includes electrostatic/electrochemical regulation [21,22]. This facilitates flexible memory effects, diverse synaptic plasticity, and various conductance modulation behaviors [22,23,24,25]. EGTs exhibit numerous advantageous properties: low operating power, operational simplicity, good reliability, decoupled read/write operations, tunable ion dynamics timescales, and multi-gate configurations [21,26,27]. These attributes position them as robust solutions for designing efficient neuromorphic circuits. Due to their distinctive ion-coupled characteristics, EGTs have successfully emulated a spectrum of neuromorphic functions, including short-term and long-term synaptic plasticity, synaptic learning rules, conditioning, and pattern recognition [23,28,29,30,31,32,33,34]. Furthermore, inspired by the human perceptual system, a sophisticated, multimodal synergistic learning network integrating sensory inputs to enhance higher cognitive functions abilities like information integration, recognition, reasoning and imagination. And the integration of EGTs with various artificial receptors has yielded biomimetic perceptual systems [26,34,35,36]. These systems demonstrate capabilities such as vision, touch, and audio-visual fusion, outlining novel design strategies for constructing artificial perception platforms [37].

This review comprehensively summarizes recent research advances in EGTs for synaptic and neuronal device applications. Specifically, we discuss the operational mechanisms of EGTs and detail their developmental progress concerning electrolyte materials, device architectures, and external field modulation. Additionally, a primary focus is the classification and description of electrolyte materials. Finally, we provide a brief discussion on future developments and the challenges that lie ahead.

## 2. Materials of Electrolytes for EGTs

In EGTs, the electrolyte serves a pivotal and distinctive function, entirely replacing the solid-state gate dielectric layer characteristic of conventional field-effect transistors [38,39,40]. This critical component is not merely an insulator but possesses significant ionic conductivity. The unique gating mechanism, which based on ionic motion, enables novel technological pathways for applications such as low-power electronics, biosensors, neuromorphic computing, and flexible/wearable electronics [15,21,41]. The physical and chemical properties of the electrolyte significantly influence the stability and functionality of EGTs. To achieve tailored control over EGT performance, diverse electrolyte materials have been engineered to address specific operational requirements. This section discusses the structural characteristics, development trajectory, and application contexts of three classes encompassing six distinct electrolyte materials. Key attributes of these six electrolytes including thickness, specific capacitance, cut-off frequency, operating voltage, and working temperature have been summarized in Table 1 [42,43]. In Table 1, the capacitance values of EGTs across different materials are all in the μF cm^−2^ range. Moreover, a thicker dielectric layer results in longer switching times and lower cut-off frequencies. The operating voltage and working temperature are the primary factors that distinguish various types of electrolyte-gated transistors.

### 2.1. Liquid Electrolytes

The foundational demonstration of electrolyte gating traces back to 1955 when researchers leveraged germanium (Ge) semiconductor substrates in conjunction with aqueous solutions to modulate carrier transport [44]. This seminal experiment established the principle of ionic charge accumulation at semiconductor–electrolyte interfaces, yet it lacked the functionality of a typical transistor. Until 1970, Bergveld pioneered the first operational transistor employing the NaCl aqueous solution electrolyte as the gate dielectric [45]. This breakthrough device integrated an aqueous electrolyte with a thermally grown SiO_2_ layer functioning as a series dielectric capacitor within a silicon MOSFET architecture. Recognized as the pristine ion-sensitive field-effect transistor (ISFET), its innovative design enabled direct sensing of ionic concentrations in physiological environments, thereby catalyzing decades of research into EGTs for bioelectronic applications [46,47,48].

**Figure 1:**
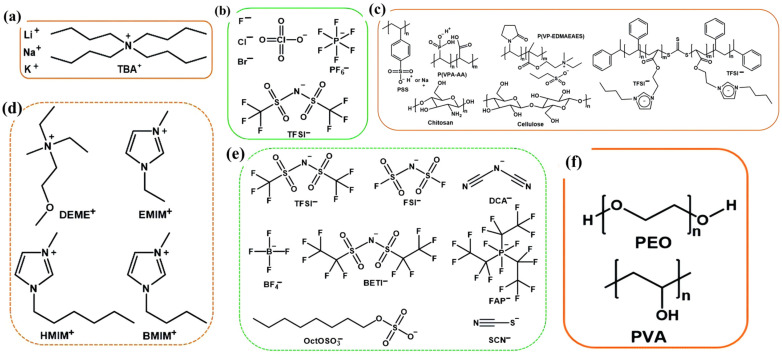
Representative liquid electrolyte chemical structures. (**a**) Cations and (**b**) anions of salts typically used in salt electrolytes. (**c**) Polymer electrolytes. (**d**) Cations and (**e**) anions of ionic liquids (ILs) typically used in electrolytes. (**f**) Polyelectrolytes [57].

Following sustained technological evolution, currently developed aqueous electrolytes incorporate diverse salts optimized for stability and biocompatibility. Predominant compounds include lithium bis (trifluoromethanesulfonyl)imide (LiTFSi), sodium chloride (NaCl), potassium perchlorate (KClO_4_), potassium hexafluorophosphate (KPF_6_), and potassium chloride (KCl), each exhibiting distinct ion transport properties as illustrated in Figure 1a,b [49]. Among these, biocompatible electrolytes such as NaCl, KCl, and phosphate-buffered saline (PBS) have become indispensable in EGTs targeting physiological signal detection, neural bio-interfacing, and continuous health monitoring [49,50]. The dominance of organic electrochemical transistors (OECTs) in this domain stems from their unique ability to transduce ionic fluxes into electronic signals within aqueous environments [41,51,52,53]. Consequently, rigorous mechanistic investigations into salt-specific effects—including ion hydration dynamics, electrochemical doping kinetics, and interfacial double-layer formation—have profoundly advanced OECT performance metrics such as transconductance, switching speed, and operational stability [53]. Progress in OECT design has concurrently driven innovations in semiconducting polymers [54]. Researchers have synthesized novel materials featuring hydrophilic side chains (e.g., ethylene glycol, oligoether) grafted onto conjugated backbones to facilitate ion penetration and volumetric doping [55,56]. These modifications yield high-performance p-type and n-type polymers with enhanced aqueous stability, including derivatives of polythiophenes (PTh), polyethylenedioxythiophenes (PEDOT), and diketopyrrolopyrroles (DPPs). Nevertheless, electrolyte composition remains a critical performance determinant across both hydrophobic archetypes (e.g., regioregular, P3HT) and hydrophilic analogs.

**Figure 2:**
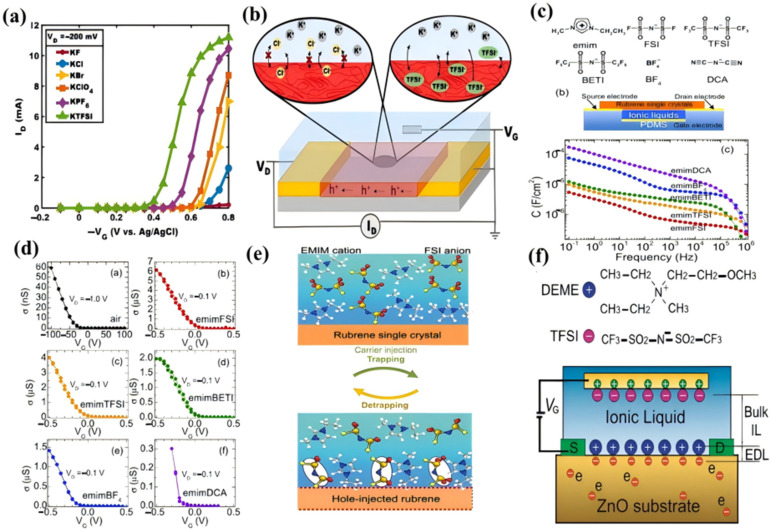
(**a**) Representative transfer curves of P3HT OECTs with six different aqueous electrolytes [58]. With permission from the American Chemical Society. (**b**) Schematic illustration showing that Cl^−^ is less intercalating than TFSI^−^ [58]. With permission from American Chemical Society. (**c**) Structure of an EDLT based on an organic single crystal semiconductor and an IL as gate dielectric (top) and capacitance of the indicated ILs as a function of frequency measured by impedance technique (bottom) [60]. With permission from Royal Society of Chemistry. (**d**) Transfer characteristics of EDLTs with an air gap, [EMIM][FSI], [EMIM][TFSI], and [EMIM][BETI] as the gate dielectric (VD = −0.1 V) [60]. With permission from the Royal Society of Chemistry. (**e**) Schematic of the carrier trapping and detrapping dynamics by the FSI anions in the first IL layer [61]. With permission from the American Chemical Society. (**f**) The structure of a ZnO field-effect transistors (FETs) [62]. With permission from Wiley-VCH Verlag.

The chemical identity of anions and cations governs ion permeability, electrochemical window, and doping efficiency through steric, electrostatic, and solvation effects. For example, to study anion-specific influences on mixed conduction in polymer–electrolyte, Ginger et al. prepared a series of poly(3-hexylthiophene) (P3HT)-based OECTs using 20 mM potassium salts with varying anions (F^−^, Cl^−^, Br^−^, ClO_4_^−^, PF_6_^−^, TFSI^−^) [58]. Their data revealed pronounced anion-dependent modulation of drain current (ID) and threshold voltage (VT), with maximum ID increasing and VT decreasing monotonically across the series: F^−^ < Cl^−^ < Br^−^ < ClO_4_^−^ < PF_6_^−^ < TFSI^−^ (Figure 2a). In contract, bulkier anions (PF_6_^−^, TFSI^−^) exhibited superior faster ion diffusion coefficients and doping efficiency in P3HT compared to smaller anions (Cl^−^, ClO_4_^−^), attributed to their lower hydration numbers and reduced effective ionic radii in solution (Figure 2b) [58]. This phenomenon arises because large-sized, weakly hydrated anions shed their solvation shells more readily during polymer insertion. Subsequent studies with hydrophilic poly(3-[[2-(2-methoxyethoxy)ethoxy]methyl]thiophene) (P3MEEMT) confirmed accelerated anion injection kinetics [59], where ethylene glycol side chains reduce the energy barrier for ion partitioning by establishing hydrated ion–transport pathways.

Collectively, these findings establish that anion selection fundamentally dictates p-type OECT characteristics—particularly VT—independent of the semiconductor’s inherent hydrophobicity [58]. Larger, less polarizable anions with low surface charge density require diminished thermodynamic driving forces for polymer penetration due to their chaotropic nature, which disrupts local water structure and favors ion–polymer interactions over ion–solvent binding. This behavior emerges from competitive equilibria between ion hydration energetics (described by the Hofmeister series) and semiconductor doping kinetics. Consequently, chaotropic anions (e.g., TFSI^−^, ClO_4_^−^) demonstrate superior doping efficiency compared to kosmotropic ions (e.g., F^−^, SO_4_^2^^−^), which maintain strong hydration spheres that impede polymer insertion. These principles now inform electrolyte engineering for high-sensitivity biosensors, neuromorphic devices, and implantable bioelectronics.

Ionic liquids (ILs), defined as salts existing in the liquid state at room temperature, offer significant advantages over traditional aqueous salt electrolytes [63,64]. These benefits stem primarily from the absence of a volatile solvent, resulting in a larger electrochemical window (typically >3 V), a low melting point (often below 20 °C), and a very high boiling point (commonly around 400 °C) [21,65]. Thus, researchers generally favor IL electrolytes over aqueous alternatives, particularly in applications where bio-compatibility is not a critical requirement. Kinds of representative IL structures in EGTs are illustrated in Figure 1. Commonly utilized cations include diethylmethyl(2-methoxyethyl)ammonium (DEME^+^), 1-ethyl-3-methylimidazolium (EMIM^+^), 1-butyl-3-methylimidazolium (BMIM^+^), and 1-hexyl-3-methylimidazolium (HMIM^+^). Frequently paired anions are bis(trifluoromethylsulfonyl)imide (TFSI^−^), bis(fluorosulfonyl)imide (FSI^−^), dicyanamide (DCA^−^), tetrafluoroborate (BF_4_^−^), bis(pentafluoroethylsulfonyl)imide(BETI^−^), tris(pentafluoroethyl)trifluorophosphate (FAP^−^), n-octylsulfate (OctOSO_3_^−^), and thiocyanate (SCN^−^) [66].

Although ILs have been widespread adoption in electrochemical mechanical actuators and electrochromic windows prior to 2007 [66], their exploration as gate dielectrics for transistors commenced that year with the work of Hebard et al., who utilized [EMIM][BETI] to gate indium oxide electric double layer transistors (EDLTs) [67]. The following year, Takeya et al. conducted investigations into single-crystal rubrene based EDLTs employing various IL electrolytes, including [EMIM][TFSI], [EMIM][FSI], [EMIM][BF_4_], and [EMIM][DCA] (Figure 2c) [60,68]. This substantial disparity in capacitance was ascribed to fundamental differences in the molecular arrangements adopted by the cations and anions within the EDL structure, coupled with variations in their slower dynamical processes. These dynamics include phenomena that the study investigated cooperative polarization in water clusters induced by an external field. Performance comparisons between rubrene-based EDLTs gated with these different ILs and conventional organic field-effect transistors (OFETS) using an air gap dielectric clearly demonstrated the operational impact of the large IL capacitance. Specifically, the high capacitance of [EMIM][DCA] facilitated the induction of an exceptionally large carrier density within the transistor channel, reaching approximately 5.3 × 10^14^ cm^−^^2^. This density was found to be about 50 times greater than the carrier densities typically achievable in devices gated with standard SiO_2_ dielectrics (Figure 2d) [69,70].

To understand how these interface effects work, researchers used three kinds of techniques: electrochemical frequency modulation, atomic force microscopy (AFM), and molecular dynamic (MD) simulations. These studies indicated that the first layer of the ionic liquid (IL) next to the electrical charges introduced into the rubrene surface gradually becomes more organized over time [61]. This structuring process involves ion reorientations and electrostatic stabilization, leading to the formation of a distinct patchwork structure (Figure 2e). In this stable configuration, negatively charged oxygen (O) and fluorine (F) atoms of anions like FSI^−^ orient themselves towards the positively charged rubrene surface, moving closer and facilitating the gradual trapping of hole carriers (Figure 2e). Furthermore, applying a large positive gate voltage deformed this structured ionic liquid (IL) layer, enabling near-instantaneous recovery of IL-induced bias stress via hole carrier detrapping. Beyond these interfacial dynamics and their impact on carrier density and trapping, the inherent liquid nature of ILs provides another key advantage: it enables effective gating of unconventional device or channel architectures where achieving conformal contact using solid dielectrics would be challenging or impossible [57,71,72,73]. In addition, In 2009, utilizing the EDL gating mechanism, ZnO field-effect transistors (FETs) generated a distinctive patchwork structure (Figure 2f) [62]. This enabled the induction of high carrier densities of 10^14^~10^15^ cm^−2^ in the ZnO channel, surpassing the 10^12^~10^13^ cm^−2^ range achievable with traditional SiO_2_ dielectric layers. This breakthrough validated the underlying mechanisms of these interfacial effects at the ILs interface (Figure 1e), achieving transformative advances in carrier density and interfacial performance for unconventional ZnO FET devices. It thereby established a foundation for subsequent exploration of synergistic EDL and doping engineering strategies. Building on this foundation of ionic control, the excellent ionic dynamics of the ionogel gate electrolyte further enable the device to demonstrate long-term memorization of excitatory chemical stimuli [74]. Interestingly, this system can also simulate chemical synaptic functions in the biological olfactory system.

### 2.2. Polymer Based Electrolytes

Typical insulating polymers do not conduct electronic charge, so they cannot form EDLs under applied voltage bias. Consequently, these materials are typically excellent gate dielectrics for various transistors architectures [27,75,76,77]. Ions must be incorporated into or added to the polymer structure to enable polymer electrolytes. As the conduction mechanism involves ion diffusion facilitated by segmental motion within the polymer matrix, polymer electrolytes exhibit excellent flexibility and tunable ionic conductivity. They are bendable, compatible with flexible substrates, making them highly attractive for flexible electronics. Figure 1c illustrates representative polymers employed within this family.

Polymer electrolytes typically consist of salts dissolved in ion-coordinating polymers (Figure 1e). The most extensively studied coordinating polymer in this family is poly(ethylene oxide) (PEO). PEO/perchlorate salts (AClO_4_, A = Li, K, Cs, etc.) are common polymer electrolytes [78,79,80]. The unpaired electron on oxygen atoms within the PEO chains coordinate with metal cations. Due to the flexibility of PEO chains, metal cations couple ion migration to the motion of the PEO backbone. Other polymers, including poly(vinyl alcohol) (PVA) and polycarbonates (PCs), have also been utilized (Figure 1c) [81,82,83,84]. LiClO_4_ is the most prevalent in polymer electrolytes [82,85,86,87,88], and in Figure 1a,b, LiC1O_4_ are the salts commonly used in aqueous electrolytes.

**Figure 3:**
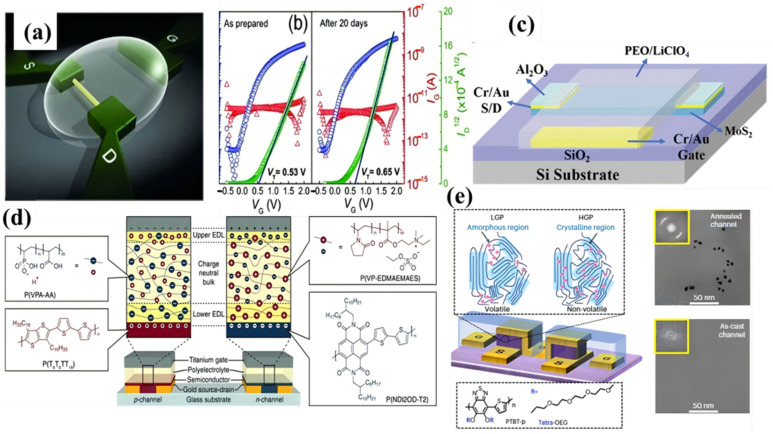
(**a**) Schematic of ZnO nanowire EDLT with inkjet-printed PEO/CsClO_4_ electrolyte dielectric [94]. With permission from Wiley-VCH Verlag. (**b**) ZnO EDLT transfer curves (IDS–VGS, VDS = 1V): fabricated vs. 20 day air exposure [94]. With permission from Wiley-VCH Verlag. (**c**) Laterally coupled 2D MoS_2_ synaptic transistor (PVA:H3PO4 electrolyte) [95]. With permission from Institute of Electrical and Electronics Engineers Inc. (**d**) The structure of complementary polyelectrolyte-gated EDLTs: (i) p-WSe_2_, (ii) n-ZnO, (iii) shared PEI:LiClO_4_ dielectric [96]. With permission from Wiley Blackwell. (**e**) Ion dynamics in volatile/non-volatile modes; Bottom: PTBT-p chemical structure and Cryo-EM images of as cast vs. 200 °C annealed films [97].

The first reported use of a polymer electrolyte as a transistor gate dielectric dates to 1987, which feature a side-gate transistor geometry by using LiCF_3_SO_3_/PEO as the polymer electrolyte and P3HT as the semiconductor [89,90]. Polymer electrolytes’ solid-state structure facilitates efficient circuit integration and enhanced stability compared to liquid electrolytes, while retaining geometry suitable for EDL formation [91,92,93]. Polymer electrolytes are prepared by dispensing a solution of polymer and salt onto the region of interest and drying it. Notably, the rheological properties of these solutions can be tuned and controlled to a greater extent than those of aqueous and IL electrolytes due to the polymer component. This makes them more suitable for various processing techniques such as inkjet printing, spin coating, and aerosol jet printing. Figure 3a demonstrates a high-performance EDLT fabricated using inkjet-printed polymer electrolyte dielectrics and single-crystalline ZnO nanowire semiconductors [94]. These ZnO EDLTs operate at low voltages (≤2 V) with an on/off ratio of 10^7^. Furthermore, as shown in Figure 3b, the transfer characteristics exhibit minimal change (e.g., ΔVt ≈ 0.2 V) after 20 days of storage in air. Printing a gate electrode onto a liquid electrolyte would be impractical, but becomes feasible with solid polymer electrolytes. Figure 3c demonstrates a synaptic transistor based on laterally coupled 2D MoS_2_, fabricated using a poly electrolyte derived from PEO and LiClO_4_ [95]. This device exhibits a pronounced EDL effect, enabling operation at low voltages (1 V) while achieving a high on/off current ratio on the order of 10^5^.

Beyond polymer electrolytes, polyelectrolytes constitute another category within polymers. Polyelectrolytes are polymers containing ionic or ionizable groups (Figure 1f) [98,99]. Representative polyelectrolytes used in EGTs can be categorized as polyanions or polycations. Polyelectrolytes are often generated by polymerizing one ionic species of an IL. Polyelectrolytes are commonly adopted as gate electrolytes in EGTs to achieve rapid switching kinetics. This capability proves particularly beneficial in OEGTs employing polymeric semiconductors [100]. Notably, organic electric-double-layer transistors (OEDLTs) utilize polyanionic and polycationic electrolytes specifically for n-type and p-type devices [96]. Strategic polyelectrolyte selection ensures compatibility with the semiconductor, effectively blocking mobile ion penetration into the organic channel. As exemplified in Figure 3d, gate modulation in OEDLTs employs P(VPA–AA) for polyanion control and P(VP–EDMAEMAES) for polycation regulation. Characterization reveals these polyelectrolytes enable swift gating responses in EDLTs while inducing negligible electrochemical doping. Contrastingly, OECT architectures leverage fundamentally distinct operating principles, as demonstrated in prior work [97]. Here, anions (e.g., [TFSI^−^] Figure 3e selectively infiltrate amorphous regions (volatile mode) or crystalline domains (non-volatile mode), initiating bulk redox reactions that modulate channel conductivity through electrochemical doping. By precisely manipulating electrode polarity and crystalline microstructure, the dynamic reconfiguration between multimodal sensing and non-volatile memory operations within a unified OECT platform are achieved [41,51,101]. This monolithic integration circumvents limitations inherent in heterogeneous systems, significantly reducing power requirements. Furthermore, the inherent hardware-level adaptability enables implementation of bio-inspired functions such as conditioned reflexes, positioning these systems as highly promising candidates for low-power embedded intelligence [102,103,104].

### 2.3. Inorganic Solid-State Electrolytes

Inorganic solid-state electrolytes (SSEs), encompassing crystalline ceramics, amorphous glasses, and composite systems, constitute an emergent material class with transformative potential for EGTs [105]. Their unique physicochemical profile—characterized by wide electrochemical stability windows (>3 V), exceptional thermal stability (>200 °C), high bulk ionic conductivity (10^−3^–10^−2^ S/cm), and ultra-low electronic leakage currents—confers critical advantages for EGTs operating under extreme environmental conditions, elevated temperatures, or integrated with conventional inorganic semiconductor platforms. These properties stem from robust ionic lattices with high activation energies for electronic transport, effectively decoupling ionic and electronic conduction pathways. Unlike liquid or polymeric electrolytes, SSEs eliminate the evaporation of solvent concerns while enabling precise interfacial engineering essential for nanoscale device integration.

**Figure 4:**
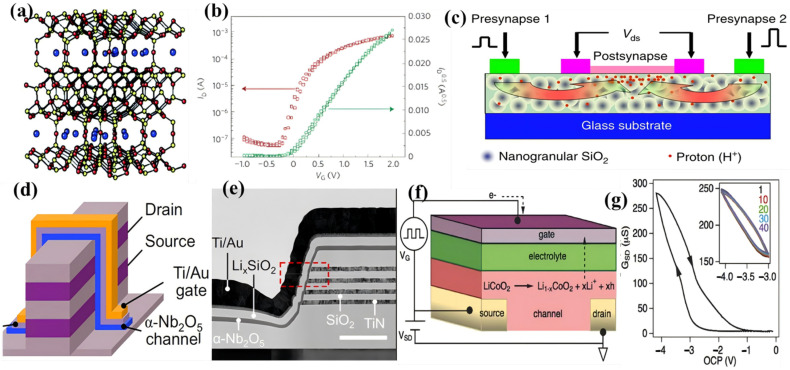
(**a**) Crystal structure of sodium beta-alumina (SBA), where blue represents the mobile sodium ions and red and yellow represent the oxygen and aluminum atoms, respectively [110]. With permission from Springer Nature. (**b**) Transfer characteristics of a ZTO transistor fabricated on an ITO glass substrate with SBA as the dielectric [110]. With permission from Springer Nature. (**c**) Schematic image of a laterally coupled synaptic transistor with two in-plane gates based on nanogranular SiO_2_ electrolytes [112]. (**d**) The structure of the $\alpha -{\mathrm{Nb}}_{2}O_{5}$ V-EGT [113]. (**e**) The cross-sectional TEM image of the $\alpha -{\mathrm{Nb}}_{2}O_{5}$ V-EGT and the TEM image of a single V-EGT highlighted by the red dotted box [113]. (**f**) Schematic of a synaptic transistor with Li1-xCoO_2_/LiPON [114]. With permission from Wiley Blackwell. (**g**) Source and drain conductance as a function of VG, with VD = 100 Mv [114]. With permission from Wiley Blackwell.

Early EGT architectures predominantly employed amorphous oxide semiconductors (AOSs) such as InGaZnO (IGZO), ZnO, and In_2_O_3_ as channel materials, typically deposited via sputtering or solution processing [106,107,108]. While these offered reasonable carrier mobility, their performance was constrained by the properties of electrolyte. Initial investigations into inorganic SSEs focused on sodium beta-alumina (β-Na-Al_2_O_3_, SBA), leveraging its crystalline superlattice structure for rapid Na^+^ conduction (Figure 4a) [109,110]. SBA precursor gels were spin-coated onto Si or ITO/glass substrates, forming dielectric films with high volumetric capacitance (>1 $\mu F/{\mathrm{cm}}^{2}$). When integrated into EDLTs, SBA electrolytes enabled ZnO-doped SnO_2_ (ZTO) devices with on/off ratios exceeding 2 × 10^4^ in Figure 4b. A significant advancement over earlier organic dielectrics. Another work utilized plasma-enhanced chemical vapor deposition (PECVD) to synthesize nanogranular SiO_2_ proton conductors at room temperature. These exhibited proton conductivities ≈ 10^−4^ S/cm through Grotthuss mechanism transport along hydroxylated surfaces [110,111,112].

A breakthrough emerged from the integration of phosphorus-doped nanogranular SiO_2_ with ZnO channels to fabricate laterally coupled IZO synaptic transistors in Figure 4c [112]. This architecture exploited proton-coupled interfacial dynamics: the application of gate voltage pulses (VG) induced proton migration across the SiO_2_ electrolyte, modulating the ZnO channel conductivity via EDL effects. Crucially, this system demonstrated biomimetic excitatory postsynaptic currents (EPSCs) with spatiotemporal signal processing capabilities. EPSCs generated by independent presynaptic inputs (Pre1 and Pre2) underwent nonlinear summation at the postsynaptic terminal—emulating dynamic logic operations essential for neuromorphic computation. This work established foundational artificial synapse networks and validated inorganic proton conductors as viable platforms for neuromorphic hardware [115].

Recent innovations have expanded the SSE repertoire to lithium-based systems. Xu et al. demonstrated vertical EGTs (vEGTs) incorporating ≈ 44 nm physical vapor-deposited LixSiO_2_ electrolytes (Figure 4d,e). [113] Mobile Li^+^ ions modulated Nb_2_O_5_ channel conductivity under gate bias, achieving all solid state neurotransistors with low operation voltages (<1 V). This vertical design allows the channel thickness to be less than 10 nm and the electrolyte thickness to be less than 20 nm, and the design capitalizes on the high Li^+^ mobility in amorphous silicates, making them particularly suitable for large-scale, low-cost fabrication, and enabling energy-efficient neural network implementation. Concurrently, cross-disciplinary insights from solid-state battery research have accelerated EGT development. Lithium phosphorus oxynitride (LiPON) electrolytes—renowned for their electrochemical stability and exceptional electronic insulation, have enabled dual-functionality devices. Beyond serving as Li-ion battery separators, LiPON facilitates gate-controlled ion injection in ECTs, demonstrating seamless integration of energy storage and computing functionalities [116].

The most transformative advancement lies in nonvolatile redox transistors (NVRTs) employing Li-intercalation channels [117,118,119]. As exemplified by Li-ion synaptic transistors (LISTA), in Figure 4f Li_1−x_CoO_2_ channels undergo reversible conductance modulation via electrochemical extraction/insertion of Li^+^ ions [116]. This mechanism fundamentally differs from resistive switching devices reliant on filament formation or phase transitions. The intercalation process preserves structural integrity while enabling analog conductance states with unprecedented linearity in Figure 4g (ΔG/G ≈ −0.0025) and low programming noise. Critically, the low activation barrier for Li+ diffusion (E ≈ 0.25 eV) permits ultra-low-energy switching <10 aJ every operation, which makes progress towards biological synaptic efficiency. NVRTs achieve this through concerted ion–electron transfer at electrode/electrolyte interfaces, where each intercalated Li^+^ dopant contributes a hole to the CoO_2_ sublattice, inducing an insulator to metal transition spanning six orders of magnitude in conductivity [114,120].

Future development of SSEs hinges on addressing key challenges: (1) Enhancing Li^+^ diffusivity in SSEs through crystalline engineering or composite design to enable μs-scale switching; (2) Mitigating interfacial degradation at semiconductor/SSE junctions via ultrathin buffer layers; (3) Scaling channel thickness below 20 nm to reduce operating voltages to <100 mV; and (4) Developing monolithically integrated crossbar arrays with selector-less operation. Advances in atomic-layer deposition and low-temperature processing show promise for fabricating these nanoscale heterostructures. As SSE chemistries evolve beyond Li^+^/H^+^ conductors [121] (e.g., Na^+^, Mg^2+^, O^2−^), they will unlock new EGT paradigms for extreme-environment computing, ultra-low-power neuromorphics, and multifunctional bio-interfaced systems.

## 3. Configuration of EGTs

The origins of EGTs can be traced back to the 1950s. EGTs share a fundamental structure with conventional FETs, comprising three key components: source/drain/gate electrical contacts, a gate charge-insulating layer (dielectric), and a charge-carrying channel formed by conductor, dielectric, and semiconductor materials [122,123,124,125]. The distinct feature of EGTs lies in their utilization of an electrolyte as the gate dielectric. This configuration endows EGTs with exceptional ion/electron coupling behavior and unique ionic relaxation capabilities, enabling the generation of extremely high electric fields and high-density charge carrier concentrations. Consequently, EGTs exhibit significantly reduced power consumption [126].

**Figure 5:**
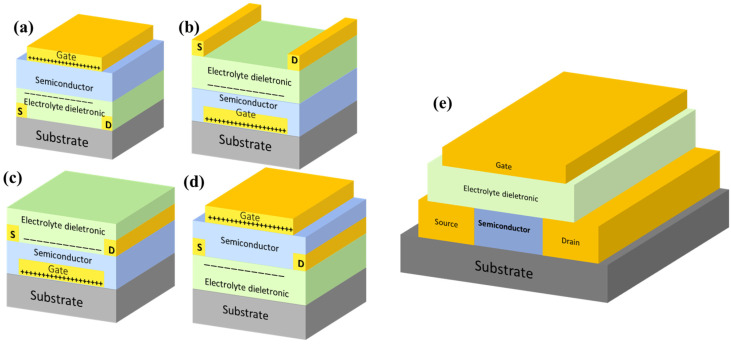
(**a**) EGTs structural configuration:top-gate bottom-contact (TGBC). (**b**) EGTs structural configuration:bottom-gate top-contact (BGTC). (**c**) EGTs structural configuration:bottom-gate bottom-contact (BGBC). (**d**) EGTs structural configuration:top-gate top-contact (TGTC). (**e**) Typically schematic diagram of EGts structure.

Based on the relative arrangement of the electrical contacts and other components, EGTs adopt various structural configurations (Figure 5a–d), namely: top-gate bottom-contact (TGBC), bottom-gate top-contact (BGTC), bottom-gate bottom-contact (BGBC), and top-gate top-contact (TGTC). Among these, the TGBC and BGTC configurations are the most prevalent, demonstrating superior characteristics compared to the BGBC and TGTC structures.

In the typical TGBC structure, metals serve as conductors, while undoped elemental silicon, metal oxides, or organic materials function as the electrolyte. Device performance is evaluated by measuring the source-drain current (conductance) under applied electric fields (bias voltages). This involves applying a bias voltage (Vd) between the source and drain electrodes, or a gate voltage (VG) between the source and gate electrodes.

The performance of thin-film transistor (TFT) devices, including EGTs, is commonly assessed based on key metrics: conductivity, threshold voltage, turn-on voltage, and the on/off current ratio. Carrier mobility under different operating conditions, a critical indicator (μ) of device performance, can be calculated using Equations (1) and (2).

(1) $$I_{D}=\mu C_{i}\frac{W}{L}[(V_{G}-V_{T})V_{D}-\frac{V_{D}^{2}}{2}]$$

(2) $$I_{D}=\mu C_{i}\frac{W}{2L}{\left(V_{G}-V_{T}\right)}^{2}$$

The selection of the appropriate drain current ($I_{D}$) model depends on $\left|V_{G}-V_{T}\right|$. Formula (1) is employed under linear operation conditions, applicable when $\left|V_{G}-V_{T}\right|$ significantly exceeds the drain voltage $V_{D}$. Conversely, Formula (2) is utilized to calculate $I_{D}$ in the saturation regime, corresponding to conditions where $\left|V_{G}-V_{T}\right|$ is less than $V_{D}$. Within these formulas, μ denotes the field-effect mobility, $V_{T}$ represents the threshold voltage, W is the channel width, and L signifies the channel length [127,128,129,130]. A schematic representation of a typical EGT structure is provided in Figure 5e. Upon application of an external electric field, ions within the electrolyte migrate towards and accumulate at the electrolyte/channel interface. This ion accumulation induces, via electrostatic coupling, a high-density accumulation of oppositely charged carriers within the adjacent channel layer. This interfacial charge separation culminates in the formation of an EDL. Two distinct operational mechanisms govern EGT behavior, defined primarily by the extent of electrochemical interaction between the electrolyte and the semiconductor channel. First, electrolyte double layer transistor (EDLT) mode: characterized by electrostatic modulation of the channel conductivity. Ions remain confined within the electrolyte, forming the EDL at the interface without penetrating into the semiconductor bulk [26,131,132]. Channel modulation occurs purely through field-effect. Second, electrochemical transistor (ECT) mode involved the electrochemical doping (or dedoping) of the semiconductor channel material. Under sufficient gate bias, ions from the electrolyte penetrate into the semiconductor layer through the highly coupled interface, inducing reversible redox reactions that alter the channel’s charge carrier density and conductivity [53,133].

### 3.1. Electrostatic Modulation

**Figure 6:**
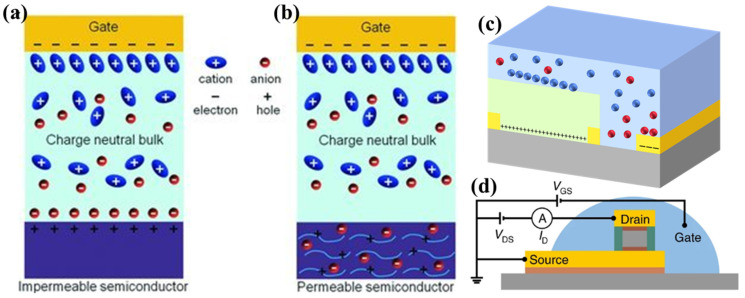
(**a**) Schematic representation of the electrostatic modulation operating mode (EDLTs) [21]. With permission from Wiley Blackwell. (**b**) Schematic representation of the electrochemical doping operating mode (ECTs) [21]. With permission from Wiley Blackwell. (**c**) The structure of the typical side field gate transistors (**d**) The structure of the vertical EGTs [132].

As schematically depicted in Figure 6a. In [21], transistors operating in the electrostatic modulation regime—where ions remain exclusively confined within the electrolyte and do not penetrate the semiconductor—are classified as EDLTs. Under steady-state conditions, the interfacial behavior at the channel/electrolyte interface is predominantly governed by the formation of an EDL. This EDL exhibits an exceptionally thin characteristic thickness on the order of ~1 nm, yet possesses an extraordinarily high specific capacitance. Leveraging this unique EDL effect at the electrolyte/channel interface, EDLTs demonstrate volatile conductance modulation at ultra-low operating voltages (<2 V).

While conventional FET models (Equations (1) and (2)) are frequently applied to characterize conductivity in EDLTs, the accurate extraction of field-effect mobility (μ) presents significant challenges. This difficulty arises primarily from the strong frequency dispersion of the electrolyte dielectric constant ($C_{i}$), a consequence of the relatively long ionic relaxation times inherent to electrolytes. Consequently, methodologies integrating gate voltage sweep frequency with the characteristic capacitance-frequency profile of the electrolyte dielectric are commonly employed to mitigate errors in mobility calculation, enabling the adoption of more precise analytical approaches.

Given these complexities, the transconductance ($g_{m}$), defined according to Equation (3), serves as a more robust and frequently reported performance metric for evaluating EGTs, particularly EDLTs [53].

(3) $$g_{m}=\frac{{\Delta I}_{D}}{{\Delta V}_{G\;}}$$

This parameter ($g_{m}$) serves as a quantitative measure of the current sensitivity of an EGT to variations in the gate voltage. Empirical evidence indicates that EGTs exhibiting higher $g_{m}$ values possess enhanced amplification capability in circuit applications [134].

### 3.2. Electrochemical Modulation

Transistors exhibiting ion-permeable and electrochemically active semiconductor channels are classified as ECTs. Under electrochemical doping conditions, anions or cations from the electrolyte accumulate at the electrolyte/channel interface and subsequently penetrate into the semiconductor channel, inducing reversible electrochemical (de)doping processes, as illustrated in Figure 6b [21].

Unlike the interfacial EDLC characteristic of EDLTs, the capacitive behavior in ECTs arises primarily from a volumetric capacitance (C*, typically exceeding 10 F cm^−3^) associated with the bulk semiconductor [41]. Critically, ECTs facilitate not only electrochemical doping but also dedoping under reverse bias polarity. Device operation fundamentally relies on these reversible doping/dedoping mechanisms, enabling non-volatile modulation of channel conductivity. These intrinsic characteristics position ECTs as highly promising candidates for emulating long-term synaptic plasticity in neuromorphic electronics.

ECTs achieve channel conductivity modulation by driving ion insertion into the channel layer, thereby altering its redox state. This mechanism confers several notable advantages, including high transconductance ($g_{m}$), low operating voltages, rapid switching speeds, and high sensitivity. Within the ECT operational framework, ions from the electrolyte modulate the bulk electronic conductivity of the semiconductor. Consequently, transconductance is often normalized by the channel geometry for more precise performance benchmarking [135]:

(4) $$g_{m1}=\frac{L}{Wd_{s}}g_{m}$$

$d_{s}$ represents the semiconductor film thickness. But it is important to note that geometry-normalized transconductance ($g_{m1}$) remains dependent on the gate voltage ($V_{G}$) in the saturation regime, as described by Equation (5). Despite this voltage dependence, the $g_{m1}$, often termed $\mu C^{*}$ in organic ECT (OECT) literature, is widely regarded as a key figure-of-merit (FOM) for these devices [53,136,137].

(5) $$g_{m1}=\mu C^{*}(V_{G}-V_{T})$$

Driven by their exceptional $g_{m}$ values and outstanding biocompatibility, ECTs have prompted significant research interest in recent years, particularly for bio-integrated electronics and neuromorphic applications. Distinct operational principles endow EDLTs and ECTs with complementary advantages and limitations. EDLTs are characterized by rapid switching speeds and reduced current-voltage (I-V) hysteresis, EDLTs are particularly suitable for integration into high-frequency logic circuits. ECTs are exhibiting superior transconductance ($g_{m}$) and high current density, ECTs serve as an ideal platform for signal amplification in applications demanding substantial current drive, such as biosensors and electrochromic elements.

Furthermore, the formation of the active interface in both EDLTs and ECTs is not constrained by rigid device/electrolyte geometries. Coupled with the inherent mechanical deformability exhibited by many electrolytes, this affords EGTs a significant structural flexibility distinct from conventional thin-film transistors (TFTs). Critically, the gate electrode placement in EGTs is highly adaptable, enabling unconventional architectures such as side gated configurations and vertically stacked architectures (Figure 6c,d) [26,132,138]. This inherent versatility positions EGTs as highly promising candidates for a diverse range of non-conventional applications, including flexible, stretchable, and bio-integrated electronics [138,139].

## 4. External Field Regulation

EGTs represent an emerging class of devices that couple ionic and electronic functionalities. EGTs’ operational mechanism, through the migration and accumulation of ions within the channel, directly emulates the variation process of the neuronal membrane potential, generating excitatory or inhibitory post-synaptic potentials in response to input signals. When the intensity of the input stimulus signal exceeds a specific threshold, the device exhibits an “all-or-none” pulsed discharge characteristic analogous to the action potential. Importantly, electrical signals applied to the gate can dynamically regulate the channel conductance state, which effectively mimics the plastic changes in synaptic connection strength—specifically, the neurobiological foundations of learning and memory: long-term potentiation (LTP) and long-term depression (LTD) phenomena. Furthermore, the relaxation time characteristics of ion movement in EGTs endow them with an inherent capability for time-dependent signal integration, enabling the processing of information dependent on the timing of stimuli. underpinning their utility in external field regulation applications. Within neuromorphic engineering, EGTs have emerged as a foundational platform for emulating synaptic functions and constructing neural network architectures. The coupled modulation of ionic and electronic processes within EGTs is achievable by subjecting them to diverse stimuli, including applied electric fields, mechanical strain, optical excitation, chemical environments, and coordinated multi-regulations. Such versatile control mechanisms underpin transformative applications in neuromorphic computing, biosensing technologies, and flexible electronic systems. Crucially, the intrinsic responsiveness of EGTs to external fields has proven pivotal in recent years for overcoming performance limitations inherent in conventional solid-state electronics.

### 4.1. Electric Field Regulation

EGTs operating under electric field control, exploit gate voltage to direct ion motion within the electrolyte. This process establishes an EDL or triggers chemical doping, ultimately modulating channel conductivity for targeted functionality. Such transistors find widespread use in logic circuits and memory devices. Presently, EGT-based logic implementations predominantly rely on unipolar inverter configurations [140,141]. These typically integrate a p-type OECT with an ion-permeable semiconducting channel, coupled with an external resistor, or a n-type EDLT employing an ion-impermeable semiconducting channel [140].

However, unipolar inverters are inherently plagued by limitations like excessive power and inadequate voltage gain, severely constraining their practical application. Complementary inverter architectures, conversely, present a compelling solution that offers low power consumption, sub-1V operation, and substantial voltage gain, thereby holding significant promise for advanced bioelectronics. A fundamental challenge, however, stems from current material constraints: achieving balanced electrical characteristics in both p-type and n-type devices fabricated exclusively from either OECTs or EDLTs remains elusive [142]. This imbalance complicates the realization of efficient EGT-based complementary circuits.

**Figure 7:**
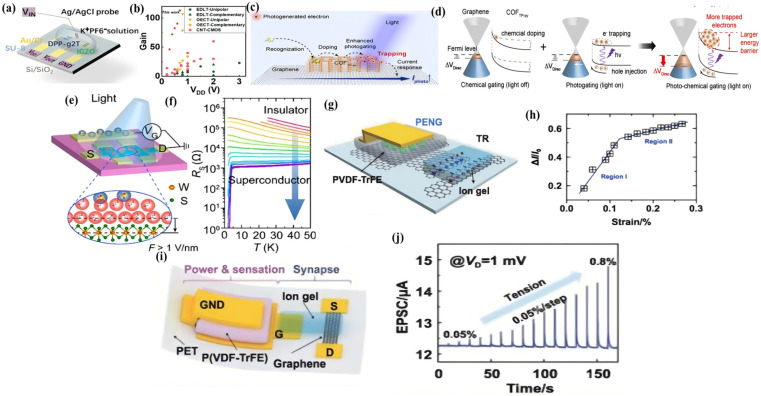
(**a**) The structure of the hybrid EGTs [143]. With permission from National Academy of Sciences. (**b**) Comparison of device voltage gain and supply voltage of this work to other literature on EGT-based inverters [143]. With permission from National Academy of Sciences. (**c**) Schematic illustration of small molecule detection by photo-enhanced chemo-transistor using photo-chemical gating mechanism [144]. With permission from American Chemical Society. (**d**) Variation in the energy band structures of COFTP-py/graphene during the photo-chemical gating [144]. With permission from American Chemical Society. (**e**) The structure of an EDLT of monolayer WS_2_ exposed to light illumination [145]. With permission from American Chemical Society. (**f**) RS Temperature dependence with various exposure times [145]. With permission from American Chemical Society. (**g**) The mechanism underlying strain sensing in the piezopotential gated [146]. With permission from Wiley Blackwell. (**h**) Sensitivity characteristics of the piezopotential gated strain sensor [146]. With permission from Wiley Blackwell. (**i**) The structure of piezotronic graphene artificial sensory synapse [147]. With permission from Wiley-VCH Verlag. (**j**) EPSC under different tensile strains [147]. With permission from Wiley-VCH Verlag.

To circumvent this hurdle, researchers devised an innovative approach by integrating a p-type OECT (utilizing the organic semiconductor polymer DPP-g2T as its active layer) with a n-type EDLT (featuring the inorganic metal oxide IGZO, In-Ga-Zn-O, as its active layer), exploiting their mutually compatible performance attributes. By harnessing the exceptional tunability of the n-type EDLT, a high-performance flexible complementary circuit was engineered using this hybrid EGT configuration (Figure 7a) [143]. This complementary design yielded a circuit delivering an exceptional voltage gain (~113) at a minimal supply voltage of 0.7 V, coupled with an ultra-low static power consumption of merely 15 nW. These metrics directly address the essential demands for EGT complementary circuits: minimal power usage, low-voltage drive, and high gain (Figure 7b) [143].

Moreover, two kinds of functional logic gates were successfully fabricated on both rigid and flexible substrates. These gates exhibit precise logic operation at driving voltages down to 0.2 V and retain stable output characteristics even when subjected to bending radii as tight as 1 mm. Demonstrating practical bio-sensing utility, the complementary circuit was employed for electrooculogram (EOG) signal acquisition, enabling tracking of vertical and horizontal eye movements. By leveraging the circuit’s inherent signal amplification (factor of ~20), the weak ~1.5 mV EOG signals were effectively boosted to ~30 mV. This capability highlights the considerable potential of such circuits for advancing wearable biosensor technologies and human–machine interface systems [148,149].

Based on these breakthrough regulation capabilities, EGT-based complementary circuits unlock transformative potential for next-generation bio-interfaced systems. Crucially, their ultra-low power operation and high signal gain enable the construction of complete, on-site signal conditioning chains. This capability is paramount for directly modulating and processing ultra-weak, noisy physiological signals (like neural spikes or biopotentials) at the point of acquisition, without relying on energy-intensive external amplification. The inherent flexibility of these circuits further allows their seamless integration into conformal bio-interfaces, facilitating real-time, closed-loop regulation of biological activity. Ultimately, this synergistic signal regulation, conditioning, and actuation capability, which all achievable with minimal power can overhead on flexible substrates. Because of this capability, EGT complementary circuits become a core building block for creating smart, responsive, and genuinely wearable bio-electronic devices. This paves the way for more advanced ways for humans and machines to interact and enables personalized healthcare diagnostics.

### 4.2. Optical Field Regulation

Optical field regulation presents an alternative and potent modulation strategy for EGTs. Integrating photoactive components or photosensitive layers allows incident light to govern ion transport dynamics and alter semiconductor band structures, facilitating synergistic interactions between photonic and ionic processes. Initial exploration of light modulation in EGTs dates back to 2014, where the work established the viability of photo-responsive EGTs and revealed the counterintuitive phenomenon termed “positive enhancement and negative suppression (Figure 7c)” [144].

Specifically, this investigation demonstrated that visible light exposure under negative gate bias (VG < 0) triggered an abrupt collapse in carrier density (up to 80%) at the LaAlO_3_/SrTiO_3_ (LAO/STO) heterointerface, concomitant with a 200-fold surge in resistance. Positive gate bias regimes exhibited conventional behavior. Crucially, light illumination amplified the gate’s efficacy by ~170, starkly contrasting the mere ~4% carrier modulation achievable via standard electric field-induced capacitive coupling alone. More detailed, Under negative gate bias, interfacial singly charged oxygen vacancies ($V_{O}^{•}$) exhibit sluggish migration, causing prolonged polarization (>2000 s). Visible light excitation, however, converts these vacancies into mobile doubly charged species ($V_{O}^{••}$), whose electric-field-accelerated migration triggers lattice expansion, suppressing interfacial conductivity and increasing resistance (Figure 7d) [144].

Based on the above study, a significant 2021 advance achieved optically controlled quantum phase transitions at cryogenic temperatures (80 K) (Figure 7e) [145]. This milestone involved operating within a frozen ILs matrix, where visible light triggered a reversible insulator-to-superconductor transition in monolayer WS_2_. This breakthrough circumvented the longstanding limitation of ionic liquid gating (EDLTs), where ion freezing at low temperatures completely impedes carrier density control. This study introduced a photon-assisted electron tunneling model under intense electric fields in Figure 7f. It posited that light illumination, combined with ultra-strong fields, enables electrons proximal to the Fermi level to tunnel into vacuum, depleting carriers. Experimentally, pre-setting an ionic-gated device into an insulating state at 220 K, followed by low-temperature (80 K) in situ optical fine-tuning of carrier density, successfully induced the superconducting transition. This ingenious opto-electro-ionic strategy thus transcended the ionic freezing barrier, unveiling a novel carrier depletion mechanism via photon-assisted field emission [145]. This work reinforces the foundational principle of optoelectronic cooperative interfacial carrier manipulation and establishes a versatile methodology for multi-field EGT modulation [150].

Today, EGTs transcend traditional limitations and unlock transformative applications in extreme environments and quantum regimes. The demonstrated ability to overcome ionic freezing barriers via photon-assisted electron tunneling fundamentally enables cryogenic quantum device operation. This paves the way for EGTs to actively modulate and probe quantum phases (superconductivity, magnetism or topological states at ultra-low temperatures, etc.) in low dimensional materials, serving as critical tools for quantum material research and potentially novel cryogenic computing paradigms. Furthermore, optical field regulation under strong electric fields offers a powerful platform for developing next-generation optoelectronic memories and neuromorphic elements capable of mimicking light-regulated synaptic plasticity. Ultimately, the synergistic opto-electro-ionic regulation strategy establishes EGTs as uniquely versatile interfaces for multi-field control at the quantum frontier, enabling novel device functionalities and advancing the frontiers of quantum electronics, cryogenic sensing, and adaptive neuromorphic systems.

### 4.3. Strain Field Regulation

Within the realm of EGTs, transistors sensitive to pressure are termed piezoelectric strain-gated transistors (PSGTs), relying fundamentally on the piezoelectric effect. This effect describes the phenomenon where certain materials develop an uneven surface charge distribution upon experiencing external mechanical pressure or vibration, resulting in a potential difference. Piezoelectric strain-gated transistors utilize stress-induced piezoelectric polarization charges to generate an internal potential at interfaces, thereby controlling electronic transport properties. The coupling between piezoelectric polarization and semiconductor transport behavior can also be used to mimic artificial sensory neurons (or afferent nerves), enabling the parallel sensing and computation of external mechanical stimuli [151]. Neuromorphic devices based on piezoelectric nanogenerator (PENG)-gated transistors have garnered significant recent attention due to advantages like self-powering capability and direct perception of external stimuli [152,153].

Sun et al. developed a PENG-gated graphene transistor [146]. This device integrates a poly(vinylidene fluoride-trifluoroethylene) [P(VDF-TrFE)]-based PENG with an ionogel-gated graphene transistor. Crucially, the bottom graphene electrode of the PENG is connected to the ionogel layer of the transistor (Figure 7g). The strain-induced piezoelectric potential from the PENG couples to the transistor’s gate, effectively modulating the Fermi level within the graphene channel and altering the carrier concentration. This study demonstrated the output characteristics of the transistor in Figure 7h, and the drain current increases with rising tensile strain applied to the PENG, exhibiting significant and rapid feedback to the piezoelectric potential. Remarkably, it maintains excellent sensitivity even under low strain conditions. Owing to its outstanding performance and novel architecture, this platform holds promise for fabricating high-performance artificial synaptic electronic devices [146].

Generally, in the human sensory system, tactile perception originates from tactile receptors detecting external mechanical stimuli and triggering changes in action potentials [154]. These action potentials are then transmitted via neurons and synapses to the central nervous system. Building upon this, a piezoelectric graphene artificial sensory synapse was proposed. This system, extending Sun’s work, comprises a PENG and an ionogel-gated transistor in Figure 7i. Upon receiving an external mechanical stimulus, the piezoelectric potential induces the formation of EDLs within the ionogel, thus modulating the carrier concentration in the channel and altering the PSC. The synaptic characteristics exhibited by the ionogel-gated transistor are influenced by different strain conditions. Figure 7j illustrates the variation in the EPSC under different strain amplitudes [147]. Information such as the magnitude and duration of the external strain can be derived from these signal changes, faithfully emulating the human tactile sensory system [154].

Electrolyte-gated transistors (EGTs) based on piezoelectric strain-gated transistors (PSGTs) are primarily employed to develop bio-inspired neuromorphic sensory systems. These devices utilize the piezoelectric effect to directly convert external mechanical stimuli (such as pressure or strain) into electrical signals. The resulting piezoelectric polarization charges regulate charge carrier transport properties within the semiconductor channel, thereby emulating the functions of afferent nerves and synapses within the human sensory system. For instance, architectures integrating piezoelectric nanogenerators (PENGs) with ionogel-gated graphene transistors can generate a self-biasing potential under mechanical strain. This potential induces the formation of electrical double layers (EDLs) within the ionogel, enabling real-time modulation of channel current (e.g., EPSC variations) and simultaneous sensing and information processing of stimulus intensity and duration. The inherent self-powering capability and high sensitivity (even under low strain conditions) render this platform ideal for constructing high-performance artificial synaptic electronic devices and biomimetic self-driven neuromorphic systems, capable of faithfully replicating the entire tactile perception pathway from signal detection to neural transmission [144,145,155].

## 5. Summary and Outlook

This review offers a comprehensive synthesis of recent breakthroughs in the rapidly evolving field of electrolyte-gated transistors (EGTs), providing both a critical analysis of current progress and a roadmap for future exploration. It commences with a succinct yet insightful overview of advancements in electrolyte materials tailored for EGTs, examining how innovations in ion-conducting media—from liquid electrolytes and polymer gels to inorganic composites—have shaped device performance metrics such as ionic conductivity, stability, and operational lifetime. Subsequently, the review delves into the fundamental operating mechanisms that distinguish EGTs, meticulously dissecting the EDLT mode, where charge accumulation at the electrolyte-semiconductor interface drives switching, and the electrochemical transistor ECT mode, which relies on redox reactions to modulate channel conductivity. This mechanistic analysis is complemented by a systematic classification of EGT developments based on external field modulation strategies, including electrostatic gating, optical field regulation, and piezopotential regulation, each highlighting unique ways to control device behavior for specific applications.

**Figure 8:**
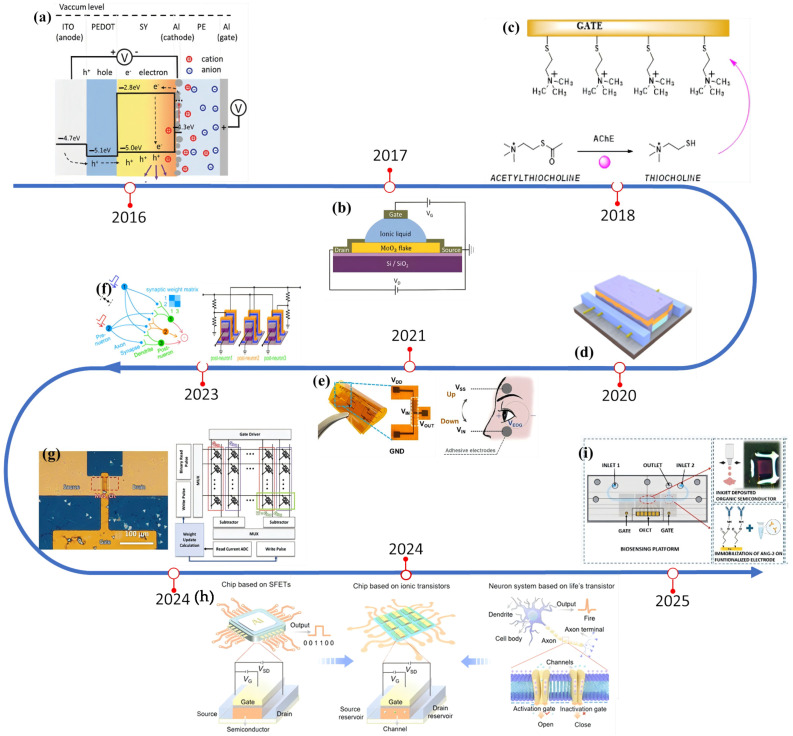
Development of EGT devices from 2016 to 2025. (**a**) The first high performance electrolyte-gated PLET [157]. (**b**) the first using 2D MoO_3_ channel material to realize the function of bionic synapse [158]. With permission from Wiley Blackwell. (**c**) Using electrolyte-gated controlled airport effect transistors (EGOFETs) monitor real-time surface reactions [159]. (**d**) The first time meets the requirements of GHz logic circuit [155]. With permission from the American Association for the Advancement of Science. (**e**) Complementary integration of p-type OECT and n-type EDLT [27]. With permission from Wiley-VCH Verlag. (**f**) A sound localization neural network [113]. (**g**) Large-scale manufacturing of EGSTs [160]. (**h**) Using high on-off ratio with ultra-low gate voltage transistors build a logic gate [156]. (**i**) Ultra-sensitive detection of tumor markers by using EGTs [161].

As follows, from the view of materials engineering, device architectures, and external control methodologies that have collectively advanced EGT technology. The review also outlines diverse application landscapes where EGTs are making inroads, while concluding with forward-looking perspectives on emerging research directions that could redefine their capabilities. Typical technological development of EGTs from 2016 to 2025 have summarized in Figure 8. Notably, the advent of carbon nanotube field-effect transistors in Nature (2020) bridged the performance gap between bio-templated devices and silicon-based electronics [155]. This breakthrough pioneered the deep integration of biological self-assembly strategies with ion transport properties of carbon nanotubes, achieving synergistic advancements in ultrafast switching speeds and biomimetic ion channels. Crucially, it established a complete technological pathway from molecular self-assembly to high-performance integrated circuits, laying the physical foundation for “ion-electron fusion computing” in the post-Moore era. Impressively, by 2021, subsequent work redefined performance boundaries for electrolyte-gated transistors (EGTs), transitioning the field from fundamental iontronic studies toward ultra-low-power, biomedically relevant electronics. It remains a cornerstone for advances in neuromorphic perception systems and flexible bio-interfacing platforms [143]. Importantly, a 2024 science study leveraged bioinspired nanofluidic design to propel ion transport to “electronic-grade” speeds while enabling programmable polarity control in ionic transistors, thus resolving the longstanding speed–polarity trade-off in conventional EGTs [156]. The construction of all-ionic logic gates not only provides a new hardware paradigm for neuromorphic computing but also marks a pivotal transition for iontronics from device-level innovation to system-level integration.

Fueled by the global surge in brain-inspired neuromorphic engineering and the exponential growth of data-intensive artificial intelligence, EGTs have emerged as a focal point in microelectronics research. Their distinctive attributes including electrostatically modifiable ion relaxation dynamics, ultra-low-voltage operation (often below 1 V), minimal power consumption (comparable to biological neurons), and precise multi-level gate tunability position them as transformative components for next-generation intelligent systems [55,162]. These characteristics are particularly advantageous for brain-like computing, where energy efficiency and analog processing capabilities are paramount, and for organic electronics, where flexibility and biocompatibility are critical. Key potential applications span a wide spectrum: emulating complex biological synaptic functions, including spike-timing-dependent plasticity and long-term potentiation; enabling large-scale parallel data processing through high-density integrated arrays that mimic neural network connectivity; facilitating energy-efficient brain-inspired computing that circumvents the power and speed limitations of the von Neumann architecture; powering portable and wearable flexible neuromorphic devices for edge computing; integrating with bio-inspired sensing systems to create intelligent “sense-and-compute” platforms; and supporting neuromorphic functional simulations in biomedical electronics, such as modeling neuronal interactions for drug discovery [16,160,163,164,165].

Despite this promise, significant challenges persist and demand targeted research efforts. In materials science, while EGT electrolytes offer a broad selection of candidates, inherent limitations—such as ionic migration hysteresis (which causes inconsistent switching) and undesired electrochemical reactions—restrict their thermal stability and maximum operating voltage relative to conventional solid-state electrolytes. This poses obstacles for applications like smart textiles and bio-mimetic robotics, which require sustained operation at moderately higher voltages and long-term stability under varying environmental conditions. Inorganic oxide electrolytes address some of these issues with superior thermal stability and higher breakdown voltages but typically require high-temperature synthesis processes, which are incompatible with the low-cost, flexible substrates used in wearable electronics. Simultaneously, SSEs suffer from inherently slow ion transport kinetics, governed by ion mobility and diffusion rates, confining their response times to the millisecond range. These challenges prevent their use in high-frequency operations and rapid switching scenarios, falling short of the sub-millisecond response times of biological synapses—a critical gap for real-time neuromorphic computing.

To overcome these limitations, the development of novel hybrid polymer electrolytes is imperative. Progress in this area can draw inspiration from adjacent fields: battery technology offers insights into enhancing ionic conductivity; supercapacitors inform strategies for improving charge storage; fuel cell research provides methods for stabilizing electrolyte-electrode interfaces; and even drug delivery systems offer approaches for controlled ion release. Cross-disciplinary knowledge from photocatalysis, actuators, dye-sensitized solar cells, and electrochromic devices may also yield innovative electrolyte designs that balance conductivity, stability, and response speed.

Structurally, high-density integration of EGTs remains underexplored, with few reports of arrays exceeding 1000 devices. Device uniformity is severely compromised by variations in thin-film deposition, where slight differences in thickness or composition across a substrate can lead to inconsistent performance. Moreover, conventional CMOS fabrication techniques often lack the precision and scalability needed to pattern EGTs with the resolution required for dense integration. Addressing these challenges hinges on merging EGT fabrication with mature semiconductor manufacturing processes. Adopting self-alignment techniques and advanced lithography—such as extreme ultraviolet (EUV) [166] or nanoimprint lithography—will be crucial for improving patterning resolution, reducing power consumption through minimized device dimensions, and enabling the production of large-scale, uniform EGT arrays.

Finally, unlocking the full potential of EGTs requires advancing beyond individual devices to system-level integration. This involves seamlessly incorporating EGTs with multi-sensory bio-sensing elements into a cohesive hardware platform that includes not only the transistor arrays but also learning algorithms, ultra-high-density neural network architectures, and functional circuit modules for signal processing. Future research must therefore adopt a more holistic approach, focusing on the collaborative optimization of complex deep learning algorithms alongside artificial multi-sensory neural systems, ensuring that hardware and software evolve in order to maximize EGTs’ transformative impact and to achieve viable commercialization [167]. To achieve viable commercialization, electrolyte-gated transistors (EGTs) must meet critical performance benchmarks: attaining ionic conductivities exceeding 10^−2^ S/cm under operating conditions to ensure sufficient current flow and rapid ion transport; achieving sub-millisecond to microsecond switching speeds for competitiveness in high-speed applications; and reaching integration densities that match or surpass current CMOS technology, potentially leveraging the inherent 3D stacking capabilities of EGT architectures.

## Figures and Tables

**Table 1:** Comparison of physical parameters of different electrolytes used for EGT.

Electrolyte Dielectric	Thickness (μm)	C (μF cm^−2^)	Cut-off Frequency (Hz)	Operating Voltage (V)	Working Temperature (°C)
Aqueous Salt Electrolytes	–	2–2000	<10^4^	~3	~100
Ionic liquids	–	1–10,000	<10^3^	~1	~400
Ion gels	0.05–400	1–200	<10^6^	~3	~300
Polymer electrolytes	0.1–500	1–100	<10^3^	~3	~300
Polyelectrolytes	0.05–100	0.2–3000	<10^4^	~3	~300
Inorganic Solid-State Electrolytes	0.02–1	0.5–1.6	<10^4^	>5	~700

## Data Availability

No new data were created or analyzed in this study. Data sharing is not applicable to this article.
